# Physical Properties of New Silica-Based Denture Surface Coating

**DOI:** 10.3390/nano15211652

**Published:** 2025-10-29

**Authors:** Kazuhiro Akutsu-Suyama, Reiko Tokuyama-Toda, Chiaki Tsutsumi-Arai, Chika Terada-Ito, Yoko Iwamiya, Zenji Hiroi, Mitsuhiro Shibayama, Kazuhito Satomura

**Affiliations:** 1 Neutron Science and Technology Center, Comprehensive Research Organization for Science and Society (CROSS), Tokai 319-1106, Ibaraki, Japan; k_akutsu@cross.or.jp (K.A.-S.); m_shibayama@cross.or.jp (M.S.); 2 Department of Oral Medicine and Stomatology, School of Dental Medicine, Tsurumi University, 2-1-3, Tsurumi, Tsurumi-ku, Yokohama 230-8501, Kanagawa, Japan; tokuyama-r@tsurumi-u.ac.jp (R.T.-T.); tsutsumi-c@tsurumi-u.ac.jp (C.T.-A.); terada-chika@tsurumi-u.ac.jp (C.T.-I.); 3 Choetsu Kaken Co., Ltd., Suehiro, Tsurumi, Yokohama 230-0045, Kanagawa, Japan; y.iwamiya@choetsu.co.jp; 4 Institute for Solid State Physics, University of Tokyo, Kashiwa 277-8581, Chiba, Japan; hiroi@issp.u-tokyo.ac.jp

**Keywords:** denture bases, *Candida albicans*, antifungal agents, biomaterials, X-ray reflectivity techniques, neutron reflectivity techniques

## Abstract

Denture stomatitis is a common issue among denture users, primarily caused by pathogenic microorganisms such as *Candida albicans* that adhere to and multiply on the denture surface. While previous approaches have focused on incorporating antimicrobial agents into denture base resins, this study introduces a novel surface coating strategy for polymethyl-methacrylate (PMMA) using hinokitiol—a natural antibacterial and antifungal compound derived from Hiba. This method enables the formation of a uniform silica–resin layer containing hinokitiol, achieved through a simple immersion process. Using X-ray and neutron reflectivity techniques, we discovered that a uniform silica–resin layer could form on PMMA with significant amounts of hinokitiol present. Time-dependent neutron reflectivity analysis revealed the presence of the following two types of hinokitiol molecules within the silica–resin layer: one type desorbs rapidly with weak capture near the surface, and the other desorbs slowly with strong capture near the PMMA interface, facilitated by hydrogen bonding in the silica–resin nanopores. These findings demonstrate a new mechanism for controlled release of antimicrobial agents from denture surfaces and highlight the potential of this coating technique as a practical and effective strategy for preventing denture-related infections.

## 1. Introduction

Denture stomatitis is a prevalent issue among denture wearers, predominantly caused by the adhesion and proliferation of pathogenic microorganisms such as *Candida albicans* on the denture surface [1,2,3,4]. The presence of cracks and irregularities on denture surfaces further complicates the removal of these pathogens, either mechanically or chemically, facilitating their adherence and growth [2,3,4]. Previous research has indicated that modifying the denture surface with specific coatings can effectively reduce microbial adhesion and suppress biofilm formation [5,6,7,8,9]. Moreover, the integration of antibacterial and antifungal agents directly into the denture base resin has been proven to inhibit the growth of pathogens such as *Candida albicans* [10]. Motivated by these findings, we aimed to develop a novel denture surface coating using the Choetsu coating technique, which offers several advantages over existing technologies [11,12]. Choetsu is a novel silica-based coating. First, we employed Choetsu coating agent to treat the denture surfaces. Unlike traditional silica glass, which forms a rigid structure with four siloxane bonds, our coating agent forms a loose three-dimensional network containing numerous alkyl groups within its siloxane bonds. Specifically, the methyltrimethoxysilane (MTMS) alkyl groups within this network facilitate the incorporation of various antifungal agents, potentially enhancing the antimicrobial efficacy of the coating [13]. Unlike other materials reported [5,6,7,8,9,10] so far as denture surface coating agents, Choetsu is capable of incorporating various antibacterial substances into a silica network, and by covalently bonding with the denture material surface, it remains on the surface for a long period of time. Furthermore, the coating can be achieved simply by applying it to the material surface and letting it dry, without the need for special equipment or techniques.

Antifungal drugs are used to treat pathogenic microorganisms such as *Candida* species that cause problems in the oral cavity. However, issues such as the emergence of resistant bacteria [14,15,16] and the existence of drugs that cannot be used in combination with these drugs have raised concerns, leading to the potential for alternative treatments using antifungal biomaterials [17,18,19,20,21] and naturally derived ingredients [22,23,24,25]. Hinokitiol, a naturally derived ingredient extracted from Hiba trees [26,27,28,29,30], has been reported to have anti-inflammatory, antioxidant, antibacterial, and antifungal properties [27,31,32,33,34,35,36]. Hinokitiol has also been reported to exhibit antibacterial properties against various oral bacteria [36,37,38], and is therefore used in toothpastes and oral care gels intended to prevent oral infections and bad breath [39,40,41]. Therefore, in our previous research, we incorporated hinokitiol into Choetsu coating technology. Prior research demonstrated that applying this coating, embedded with hinokitiol, to test pieces of polymethyl methacrylate (PMMA) denture base resin effectively inhibited the growth of *Candida albicans* [42]. Further analysis using Fourier transform infrared (FT-IR) and nuclear magnetic resonance (NMR) confirmed the successful coating of the PMMA surface and the integration of hinokitiol. These findings suggested that our new coating technology could be instrumental in preventing issues like denture stomatitis by modifying the surface properties of dentures [42].

Despite these promising results, the longevity of the coating and the duration of hinokitiol release from the denture surface remained uncertain. Therefore, this study aimed to investigate the thickness of the coating film, the distribution of hinokitiol within it, and the stability of hinokitiol over time. We employed X-ray (XR) and neutron reflectivity (NR) techniques, recognized for their nondestructive evaluation of the thickness, density, and roughness of thin layers [43,44]. Additionally, the use of deuterium (D) labeling techniques enhanced the neutron scattering contrast, allowing for a detailed analysis of the organic components within the samples. By using fully deuterated hinokitiol, we were able to significantly improve the visibility in neutron scattering, facilitating a thorough investigation of hinokitiol’s distribution and retention within the silica–resin thin layers over time [45,46].

## 2. Materials and Methods

### 2.1. Materials

The following materials were used without further purification. For synthesis: hinokitiol (TCI Chemicals Co., Ltd., Tokyo, Japan), deuterium oxide (D_2_O, 99.9% D, Sigma-Aldrich, St. Louis, MO, USA), Pt/C (10 wt% Pt, N.E. CHEMCAT Co., Tokyo, Japan), Pd/C (10 wt% Pt, N.E. CHEMCAT Co., Japan), 2-propanol, ethanol (EtOH), ethyl acetate, 1,4-dioxane, toluene, and chloroform (CHCl_3_) and chloroform-*d* (CDCl_3_) (FUJIFILM Wako Pure Chemical Co., Osaka, Japan). For silica–resin layer sample preparation: (heat cured) polymethyl methacrylate (FUJIFILM Wako Pure Chemical Co., Japan), silicon wafers (diameter = 5.08 cm, thickness = 2.0 mm, Crystal Base Co., Ltd., Osaka, Japan), the coating agent, a mixture of oligomers of methyltrimethoxysilane (MTMS; CH_3_Si(OCH_3_)_3_), poly(methylphenylsilane) (PMPS; [Si(CH_3_)(C_6_H_5_)O]_n_), and 2-propanol (CHOETSU KAKEN Co., Ltd., Yokohama, Japan). Ultrapure water (18.2 MΩ·cm) was produced using a deionized water system (RFU424TA, ADVANTEC, Tokyo, Japan).

### 2.2. Synthesis of Deuterated Hinokitiol (Hinokitiol-d_10_)

**Scheme 1:**
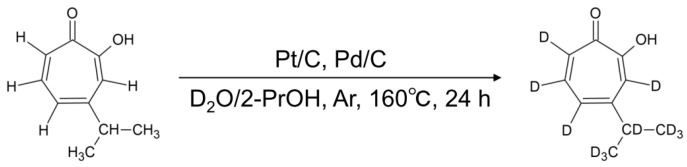
Synthesis of hinokitiol-*d*_10_.

The deuteration reaction (Scheme 1 and Scheme S1) was performed in a stainless-steel reactor (TSSR, TPR1-VSI-300, SUS316, Taiatsu Techno Corp., Tokyo, Japan) [47]. A mixture of hinokitiol-*h*_12_ (4.48 g, 27.3 mmol), Pt/C (10 wt% Pt, 1.00 g, 0.51 mmol), and Pd/C (10 wt% Pd, 0.62 g, 0.58 mmol) in a D_2_O (100 mL)/2-propanol (20 mL) solvent was loaded into the TSSR. The mixture was vacuum-degassed for 3 min to remove oxygen and then purged with argon for 10 s before sealing. The reactor was heated to 160 °C and stirred continuously for 24 h. After cooling to 20 °C, the mixture was filtered through a Celite plug to remove the catalyst and washed twice with ethanol (100 mL). The filtrate was evaporated under reduced pressure, and the crude product was purified using silica gel chromatography, eluting with ethyl acetate to yield 3.37 g (72.6%) of hinokitiol-*d*_10_. ^1^H NMR (400 MHz, CDCl_3_) *δ* 1.23 (residual signal), 2.86 (residual signal), 6.96 (residual signal), 7.27–7.35 (residual signals); ^2^H NMR (61.4 MHz, CHCl_3_) *δ* 1.19 (s), 2.86 (s), 7.06 (s), 7.18 (s), 7.26 (s), 7.42 (s). ^13^C NMR (101 MHz, CDCl_3_) δ 22.0–23.2 (m), 37.8–38.5 (m), 121.7–122.3 (m), 122.8–123.4 (m), 127.1–127.7 (m), 136.6–137.2 (m), 160.0 (s), 171.0 (s), 171.4 (s). Electrospray ionization mass spectrometry (ESI-MS), ^1^H, ^2^H, and ^13^C NMR data are available in the Supplementary Materials (Figure S1).

### 2.3. Preparation of Silica–Resin and Hinokitiol-Containing Silica–Resin Layer Samples

A preliminary test was conducted to assess the feasibility of applying a silica coating to the surface of the plastic specimen, and it was confirmed that a thin silica layer had been successfully formed on the plastic plate. Additional information is provided in the Supplementary Materials.

Before synthesizing thin silica–resin layer samples, the PMMA substrate was prepared. Silicon wafers were washed in ethanol and acetone and subsequently dried under a stream of argon gas at ambient temperature (approximately 20 °C) for 30 min. Using a spin-coater (MS-A150, Mikasa Co. Ltd., Tokyo, Japan), the thin PMMA layer was coated on the silicon wafer by spin coating a 1 wt% PMMA/toluene solution at 3000 rpm, followed by heating the sample at 110 °C for 8 h. These PMMA/Si substrate samples were used as the PMMA substrate in this study.

A silica–resin thin-layer sample (SR/PMMA/Si) was prepared on the PMMA substrate by spin coating a 10-fold diluted silica–resin solution at 3000 rpm using a spin-coater. Subsequently, the samples were cured at 80 °C for 8 h and stored in a box under low-humidity and dust-free conditions.

For the hinokitiol-containing silica–resin thin-layer sample (SR-HT/PMMA/Si), a mixture of the 10-fold diluted silica–resin solution (0.693 g) and hinokitiol-*d*_10_ (0.104 g, 0.63 mmol) was spin-coated onto the PMMA substrate at 3000 rpm using the spin-coater. Subsequently, the sample underwent a similar curing process at 80 °C for 8 h and was stored under identical conditions.

### 2.4. Neutron Reflectivity Measurement

NR measurements were performed using a polarized neutron reflectometer (BL17 SHARAKU, Ibaraki, Japan) [48]. A mercury target generated pulsed neutron beams at a frequency of 25 Hz, and the time-of-flight (TOF) method was used to measure the NR data. Disk choppers were used to tune the wavelength (*λ*) of the incident neutron beam from 2.2 to 8.8 Å for the unpolarized neutron mode. The covered scattering vector (*Q*_z_) range was 0.008 to 0.2 Å^−1^, where *Q*_z_ = (4*π*/*λ*) sin*θ* (*θ* represents the angle of incidence). Using six distinct collimating slits, a 10 mm beam footprint was continuously maintained on the sample surface. A ^3^He gas tube detector with no spatial resolution was used to gather TOF neutron data. The measurements were conducted at an ambient temperature of 20 °C. Data acquisition at the MLF was performed using the event recording method [49]. Data reduction, normalization, and subtraction were performed using the software available at BL17 SHARAKU. The Motofit software [50] was used to fit the reflectivity profiles, employing a least-squares approach to minimize deviations of the fit. The scattering length density (SLD) was evaluated using Motofit (Rev. 409). The molar composition and physical density of the sample can be used to determine the SLD, which is a nuclear property of individual atoms.

### 2.5. X-Ray Reflectivity Measurement

X-ray reflectivity (XR) measurements were conducted at the BL8S1 beamline station using a Rigaku SmartLab (Rigaku, Tokyo, Japan) diffraction apparatus with vertical scattering geometry installed at the Aichi Synchrotron Radiation Center. The synchrotron radiation was monochromatized using an Si(111) crystal monochromator. The reflected X-rays were recorded by a two-dimensional detector (PILATUS-100K, DECTRIS Ltd., Baden, Switzerland). The energy of the incident X-ray was 9.15 keV, and the measured *Q*_z_ range was from 0.028 to 0.48 Å^−1^. All measurements were carried out at 20 °C in an ambient atmosphere.

## 3. Results and Discussion

### 3.1. X-Ray Reflectivity Analysis of the SR/PMMA/Si and SR-HT/PMMA/Si Sample

To ensure clarity, we emphasize that the XR and NR samples are distinct and were prepared independently on different dates.

**Figure 1:**
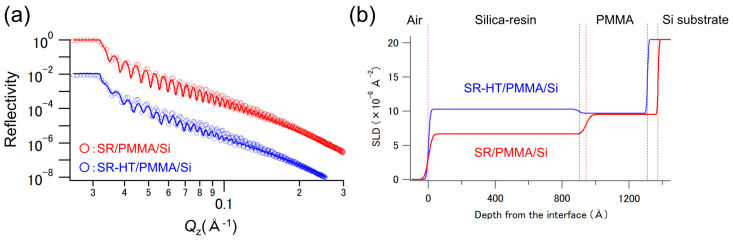
(**a**) X-ray reflectivity profiles of the SR/PMMA/Si and SR-HT/PMMA/Si samples. Circles represent experimental data, and solid lines indicate the best-fit calculated XR profiles. Profiles are vertically shifted for clarity. (**b**) X-ray SLD profiles of the SR/PMMA/Si and SR-HT/PMMA/Si samples, derived from the obtained structural parameters. Profiles are vertically shifted for clarity.

XR measurements were conducted to determine the composition of the synthesized silica–resin thin layers on PMMA substrates. Figure 1a shows the XR profiles of the air–solid reflectivity data for the SR/PMMA/Si and SR-HT/PMMA/Si samples along with their respective fitting results. Due to the presence of a naturally oxidized SiO_2_ thin layer on the surfaces of the silicon substrates, a three-layer model (SR/PMMA/SiO_2_(oxidized)/Si or SR-HT/PMMA/SiO_2_(oxidized)/Si) was employed to fit the XR profiles. The symbols in the figure represent the observed reflectivity profiles, whereas the solid lines correspond to the calculated reflectivity profiles determined from the structural models. The theoretical reflectivity profiles reproduced the experimental data across the *Q*_z_ range. The thickness and SLD of the SR thin layer were estimated to be 944 Å and 6.67 × 10^−6^ Å^−2^, respectively. These findings indicate a uniform density throughout the depth of the silica–resin thin layer, estimated at 0.99 g cm^−3^. This density closely matches the 0.925 g cm^−3^ reported in previous studies [13], indicating that the silica–resin thin layer synthesized on the PMMA substrate is of comparable quality to that produced under standard conditions. In contrast, the SR-HT thin layer exhibited a thickness and SLD of 900 Å and 10.29 × 10^−6^ Å^−2^, respectively. The increased SLD suggests that hinokitiol has been incorporated into the silica layer. Although the SLD value of the silica layer was found to be high at 10.29 × 10^−6^ Å^−2^, the corresponding density of 1.53 g cm^−3^ makes it difficult to definitively conclude from XR data alone that hinokitiol has been integrated into the silica–resin layer; however, it is plausible based on these results.

Further detailed analysis of the XR data, as shown in Figure S2, utilized a four-layer model assuming a mixed layer of silica–resin and PMMA atop the PMMA layer. Although the fitting results of this model were not significantly different from those of the three-layer model, the obtained chi-square value—a metric for determining the best fit—was lower for the four-layer model. Previous studies have reported conflicting results regarding the mixing of the layers at the interface between organic polymers and silica [51]. The compatibility of these materials may influence their mixing at the interface. Considering these findings, it is likely that a thin mixed layer of SR and PMMA was formed at the SR and PMMA interface due to the silica–resin synthesis method employed in this study.

### 3.2. Neutron Reflectivity Analysis of the SR-HT/PMMA/Si Sample

Neutron reflectometry, enhanced by deuterium labeling techniques, provides a detailed analysis of thin-layer structures. For this study, we prepared a deuterated hinokitiol-containing SR-HT/PMMA/Si sample specifically for NR experiments.

First, we examined the thin-layer structure of the SR/PMMA/Si sample using NR. The NR profiles depicted in Figure S5 (available in Supplementary Materials), confirmed the consistency of the theoretical and experimental reflectivity profiles across the *Q*_z_ range. The SLD value of the silica–resin layer was determined to be 0.6 × 10^−6^ Å^−2^, aligning with the XR results and confirming the SLD value of silica–resin in its dry state, devoid of hinokitiol.

**Figure 2:**
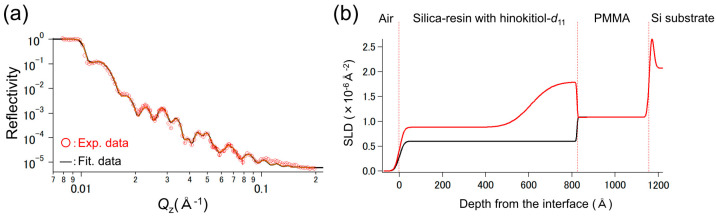
(**a**) Neutron reflectivity profiles of the SR-HT/PMMA/Si sample, with circles indicating the experimental data and solid lines the best-fit calculated NR profiles. (**b**) Neutron SLD profiles of the SR-HT/PMMA/Si sample, calculated from the obtained structural parameters. The black solid line represents the SLD profile for a silica–resin sample without hinokitiol-*d*_10_.

Figure 2a shows the NR profiles of the SR-HT/PMMA/Si sample measured 8 h post-curing. Initially, a three-layer model (SR-HT/PMMA/SiO_2_(oxidized)/Si) was applied to fit the NR data; however, adjustments to the fitting parameters did not yield satisfactory results. Consequently, unlike the XR results, a more complex four-layer model (hinokitiol-poor SR/hinokitiol-rich SR/PMMA/SiO_2_(oxidized)/Si) was used. Figure 2b illustrates the neutron SLD profiles of the SR-HT/PMMA/Si sample derived from the structural parameters. The solid black line represents the assumed SLD profile for a silica–resin sample without hinokitiol-*d*_10_. The large SLD difference between the red and black lines indicates a substantial concentration of hinokitiol within the silica–resin layer. These NR results indicate the formation of a dual-layer structure in the SR-HT layer, comprising a hinokitiol-rich layer and a hinokitiol-poor layer. If the density of the silica–resin layer is assumed to be 1.53 g/cm^3^, the expected SLD value would be approximately 0.9 × 10^−6^ Å^−2^. The actual range of SLD values from 0.9 to 1.79 × 10^−6^ Å^−2^ in the SR-HT layer supports the incorporation of hinokitiol-*d*_10_ into the silica–resin layer. The SLD profile further revealed a higher concentration of hinokitiol-*d*_10_ approximately 200 Å near the PMMA layer interface than near the surface of the silica layer. This significant hinokitiol adsorption at the SR/PMMA interface is likely due to the nanopores near this interface, which offer a structure conducive to supporting hinokitiol, or because of the high affinity between PMMA and hinokitiol, leading to its accumulation at the PMMA/siloxane interface.

### 3.3. Studies on the Hinokitiol Sustained-Release System

**Figure 3:**
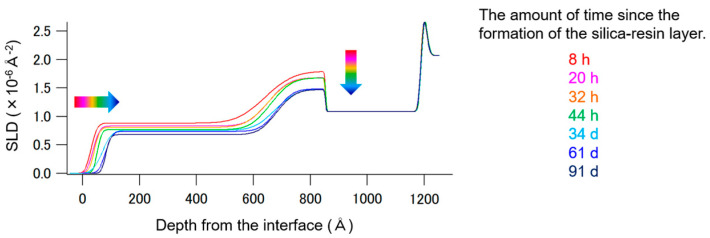
Dependence of the SLD profiles of the SR-HT/PMMA/Si sample on the time elapsed since the formation of the silica–resin layer.

We investigated the sustained-release system of hinokitiol from the SR thin layer using time-dependent neutron reflectometry. Figures S5–S10 display all time-dependent NR data, and Figure 3 illustrates the time-dependent changes in the SLD profiles of the SR-HT/PMMA/Si sample, revealing that (1) the silica–resin layer progressively thinned and (2) the SLD value of the SR-HT layer decreased over time, indicating the desorption (volatilization) of hinokitiol from the layer.

**Figure 4:**
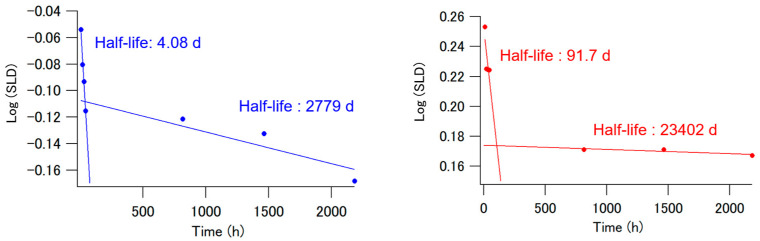
Profiles of SLD changes at the surface (**left**) and interface (**right**) of the SR-HT layer. The estimated half-life values are indicated.

Based on the time-dependent NR data (Figure 3 and Figures S6–S12), the half-life of the hinokitiol-desorption reaction was estimated. Figure 4 shows the SLD changes at the surface (200 Å) and interface (800 Å) regions of the SR-HT layer. It appears that the SR layer hosts hinokitiol species with varying affinities for silica, leading to a biexponential decrease in SLD values, which is characterized by fast and slow desorption rates. The hinokitiol-desorption reaction from the silica–resin can be modeled by the following first-order kinetic equation:

[C] = [C]_0_ exp(–*kt*) (1)

where [C] represents the concentration of hinokitiol remaining after time *t*, [C]_0_ is the initial concentration, *k* is the desorption rate constant, and *t* (h) is the elapsed time since the formation of the silica–resin layer, corresponding to the duration between the synthesis of the SR-HT/PMMA/Si sample and the NR measurement. Due to challenges in directly calculating [C] and [C]_0_ from SLD profiles, the SLD values at 8 h and *t* hours were used to approximate these concentrations. The resulting rate constants and corresponding half-life values are summarized in Table 1. The observed difference in the volatilization rate of hinokitiol between the surface of the silica–resin layer and the region near the PMMA interface is presumably attributable to its sequential evaporation, beginning from the surface. Thus, it can be said that no unusual behavior is observed in the fast hinokitiol desorption reaction. On the other hand, the hinokitiol-desorption half-lives differ by over 100-fold between species strongly bound within the silica–resin and those more loosely associated, demonstrating the substantial influence of the nano-porous structure on hinokitiol confinement and release kinetics. Notably, even the faster reaction exhibited a half-life of 4 days, suggesting a pronounced effect of the nano-structured environment.

**Figure 5:**
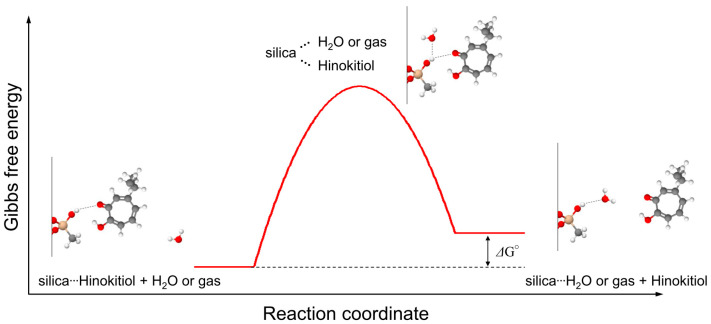
Diagram illustrating the Gibbs free energy changes for the hinokitiol-desorption reaction.

Furthermore, we elucidated the mechanism behind the strong incorporation of hinokitiol in the silica–resin. Using the estimated hinokitiol-desorption reaction rate constants from the NR data, we calculated the Gibbs free energy (ΔG°) of the hinokitiol-desorption reaction from the silica–resin thin layer. We propose that the hinokitiol-desorption reaction in the silica–resin pores involves a replacement reaction with H_2_O or gas molecules from the air, given that SiO_2_ surfaces tend to adsorb water and/or gas molecules [52,53,54,55]. The reaction mechanism is depicted in Figure 5. The Gibbs free energy for this reaction is defined as follows:

ΔG° = −RT ln(*k*) (2)

where R is the gas constant (8.314 J mol^−1^K^−1^). The calculated Gibbs free energies are summarized in Table 2.

Since the ΔG° values are positive for all reactions, these reactions are endothermic, likely to be promoted by high temperature or low pressure. Given that the heat of vaporization of water is 40.65 kJ mol^−1^ [56,57], the observed ΔG° values ranging from 10.0 to 31.1 kJ mol^−1^ suggest that hinokitiol can undergo volatilization even at ambient temperature and pressure. The differences in ΔG° between the fast and slow hinokitiol-desorption reactions are 15.9 kJ mol^−1^ at the surface region and 13.5 kJ mol^−1^ at the interface region, respectively. These values are comparable to the energy of hydrogen bonds (5–40 kJ mol^−1^). Thus, it can be proposed that hinokitiol molecules form hydrogen-bonding interactions within the silica–resin nanopores, resulting in those that form hydrogen bonds exhibiting lower evaporation rates than those that do not. Furthermore, the ΔG° differences between the surface and interface reactions, ΔG°_ss_ vs. ΔG°_si_ and ΔG°_fs_ vs. ΔG°_fi_, are 5.2 kJ mol^−1^ and 7.6 kJ mol^−1^, respectively. These values are associated with the energy associated with weak interactions, such as van der Waals forces (0.4–4 kJ mol^−1^) or hydrogen bonds. As discussed in the XR study section, the formation of a thin mixed layer of SR and PMMA at the interface could contribute to increased hydrogen-bonding interactions due to the presence of PMMA. Therefore, the variation in hinokitiol’s sustained-release properties appears to result from a combination of hydrogen bonding within the nanopores and van der Waals (or hydrogen bond) forces at the PMMA interface, with weak interaction forces of a few kJ mol^−1^ occurring at this interface.

### 3.4. Application in Dental Medicine and Stomatology

The NR results demonstrate that the silica–resin layer contracts by 5.2 nm upon hinokitiol desorption, indicating that the layer can expand and contract in response to substance adsorption and desorption. This indicates that the flexibility of the silica–resin layer may contribute to the effective adsorption of hinokitiol. Consequently, silica–resin is not only chemically stable but also sufficiently flexible, making it an excellent material for dental applications.

Furthermore, it was established that hinokitiol is effectively encapsulated within the siloxane film formed by the new silica-based coating technology on the PMMA surface. The calculated half-life of hinokitiol is approximately 4 days at the surface and 92 days at the interface. This duration is considered adequate for achieving antibacterial activity while a patient uses a single denture. Given that the actual denture coating film is several micrometers thick—substantially thicker than the thin film used in our studies—it is plausible that hinokitiol could be supported for an even longer period. Moreover, the possibility of reapplying the coating after the half-life of hinokitiol has passed allows for continuous adaptation to the patient’s needs. Our findings confirmed that hinokitiol remains on the denture surface for a sufficient duration and is released slowly and effectively.

## 4. Conclusions

In this study, we have demonstrated the formation of a uniform silica–resin layer on PMMA, incorporating significant amounts of hinokitiol. We observed a variance in the retention period of hinokitiol influenced by its depth within the surface of the PMMA coated with a new silica-based coating. Notably, hinokitiol, which is strongly bound within the silica–resin layer, exhibited an extended half-life of approximately 7.5 years. This longevity is likely due to the formation of a mixed layer of SR and PMMA at the SR-PMMA interface, which facilitates robust interactions with hinokitiol. The application of this coating on dentures could sustain antibacterial properties over an extended period, potentially addressing long-term issues such as denture stomatitis associated with the use of dentures.

## Figures and Tables

**Table 1:** Obtained desorption rate constant and corresponding half-lives for the hinokitiol desorption reaction.

	Desorption Rate Constant	Half-Lives
Fast hinokitiol-desorption reaction at the surface (*k*_fs_)	1.6 × 10^−2^ h^−1^	4.08 d
Slow hinokitiol-desorption reaction at the surface (*k*_ss_)	2.4 × 10^−5^ h^−1^	2779 d
Fast hinokitiol-desorption reaction at the interface (*k*_fi_)	7.2 × 10^−4^ h^−1^	91.7 d
Slow hinokitiol-desorption reaction at the interface (*k*_si_)	2.8 × 10^−6^ h^−1^	23,402 d

**Table 2:** Calculated Gibbs free energies of the hinokitiol-desorption reaction.

	ΔG°
ΔG° for the fast hinokitiol-desorption reaction at the surface (ΔG°_fs_)	10.0 kJ mol^−1^
ΔG° for the slow hinokitiol-desorption reaction at the surface (ΔG°_ss_)	25.9 kJ mol^−1^
ΔG° for the fast hinokitiol-desorption reaction at the interface (ΔG°_fi_)	17.6 kJ mol^−1^
ΔG° for the slow hinokitiol-desorption reaction at the interface (ΔG°_si_)	31.1 kJ mol^−1^

## Data Availability

The original contributions presented in this study are included in the article/Supplementary Materials. Further inquiries can be directed to the corresponding author.

## Supplementary Materials

The following supporting information can be downloaded at: https://www.mdpi.com/article/10.3390/nano15211652/s1, Scheme S1. Reaction scheme of hinokitiol deuteration. Figure S1. (a) ^1^H NMR (CDCl_3_, 400 MHz), (b) ^2^H NMR (CHCl_3_, 61.4 MHz), and (c) ^13^C NMR (CDCl_3_, 101 MHz) data of hinokitiol-*d*_10_. 1,4-Dioxane: deuteration rate reference, CDCl_3_ or CHCl_3_: NMR solvents. (d) ESI-MS spectra in positive mode of hinokitiol-*d* cation showing the mass distribution of the different isotopologues, which ranges from *d*_11_–*d*_15_. The distribution of the isotopologues is as follows (M^+^): 7.4%, *d*_8_; 19.8%, *d*_9_; 38.7%, *d*_10_; 34.2%, *d*_11_ (calculated deuteration level = 90.9%); Figure S2. Elemental (C, O, and Si) mapping of the test sample. Figure S3. The photograph of the SR-HT/PMMA/Si sample (left) and a schematic illustration of its structure; Figure S4. (a) XR profiles of the SR/PMMA/Si sample. The circles represent the experimental data, while the solid lines represent the best-fit calculated NR profiles. (b) X-ray SLD profiles of the SR/PMMA/Si sample calculated from the obtained structural parameters; Figure S5. (a) NR profiles of the SR/PMMA/Si sample. The circles represent the experimental data, while the solid lines represent the best-fit calculated NR profiles. (b) Neutron SLD profiles of the SR/PMMA/Si sample calculated from the obtained structural parameters; Figure S6. NR profiles of the SR/PMMA/Si sample (8 h). The circles represent the experimental data, while the solid lines represent the best-fit calculated NR profiles; Figure S7. NR profiles of the SR/PMMA/Si sample (20 h). The circles represent the experimental data, while the solid lines represent the best-fit calculated NR profiles; Figure S8. NR profiles of the SR/PMMA/Si sample (32 h). The circles represent the experimental data, while the solid lines represent the best-fit calculated NR profiles; Figure S9. NR profiles of the SR/PMMA/Si sample (44 h). The circles represent the experimental data, while the solid lines represent the best-fit calculated NR profiles; Figure S10. NR profiles of the SR/PMMA/Si sample (34 d). The circles represent the experimental data, while the solid lines represent the best-fit calculated NR profiles; Figure S11. NR profiles of the SR/PMMA/Si sample (61 d). The circles represent the experimental data, while the solid lines represent the best-fit calculated NR profiles; Figure S12. NR profiles of the SR/PMMA/Si sample (91 d). The circles represent the experimental data, while the solid lines represent the best-fit calculated NR profiles. Ref. [58] is cited in the supplementary materials.
