# Supplementary material for: Physical Properties of New Silica-Based Denture Surface Coating
Source: Nanomaterials (Basel). 2025 Oct 29;15(21):1652. doi: 10.3390/nano15211652 (PMC12610639; doi:10.3390/nano15211652)
Supplement: Supplementary file 1 [file nanomaterials-15-01652-s001.zip › nanomaterials-3947313-supplementary.pdf]

Supplementary Information

Physical Properties of New Silica-Based Denture Surface Coating

Kazuhiro Akutsu-Suyama [1,†], Reiko Tokuyama-Toda [2,†], Chiaki Tsutsumi-Arai [2], Chika Terada-Ito [2], Yoko Iwamiya [3], Zenji Hiroi [4], Mitsuhiro Shibayama [1] and Kazuhito Satomura [2,*]

[1] Neutron Science and Technology Center, Comprehensive Research Organization for Science and Society (CROSS), Tokai 319-1106, Ibaraki, Japan
[2] Department of Oral Medicine and Stomatology, School of Dental Medicine, Tsurumi University, 2-1-3, Tsurumi, Tsurumi-ku, Yokohama 230-8501, Kanagawa, Japan
[3] Choetsu Kaken Co., Ltd., Suehiro, Tsurumi, Yokohama 230-0045, Kanagawa, Japan
[4] Institute for Solid State Physics, University of Tokyo, Kashiwa 277-8581, Chiba, Japan
* Correspondence: satomura-k@tsurumi-u.ac.jp; Tel.: +81-45-580-8333
† These authors contributed equally to this work.

Table of Contents

Reaction scheme of hinokitiol deuteration 2

ESI-MS and NMR spectra of deuterated hinokitiol 2

EPMA analysis conducted on test samples 4

X-ray and Neutron Reflectivity Analysis 5

References 9

1. Reaction scheme of hinokitiol deuteration

A schematic overview of the experimental procedure and setup is provided to ensure a clearer understanding of the deuteration method for hinokitiol.

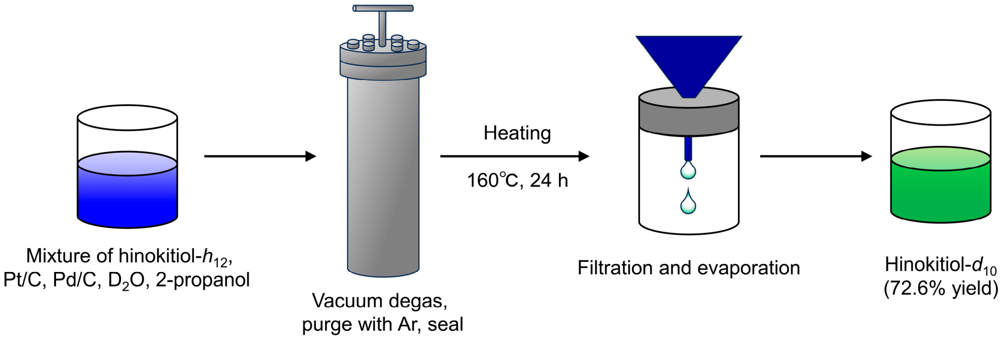

Scheme S1. Reaction scheme of hinokitiol deuteration.

2. ESI-MS and NMR spectra of deuterated hinokitiol

The ^{1}H, ^{2}H, and ^{13}C NMR spectra of deuterated hinokitiol were recorded using a 400 MHz NMR spectrometer (JEOL JMTC-400/54/JJ/YH spectrometer). The deuteration level of deuterated hinokitiol (92.2 %) was evaluated from the integral value of the ^{1}H NMR signals arising from the deuterated hinokitiol and the 1,4-dioxane internal standard.[1] Electrospray ionization mass spectra (ESI-MS, Nihon Bunko Co. Ltd., Japan) were recorded on an EXTREMA-MS-100P spectrometer.

(a) ^{1}H NMR

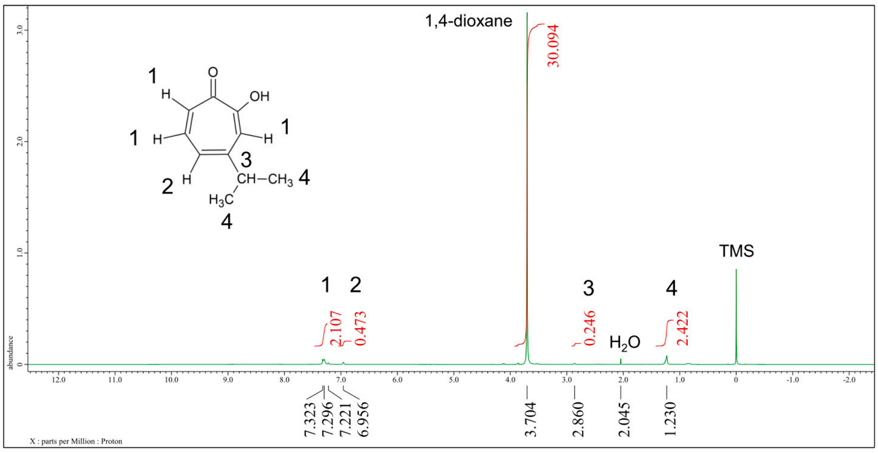

(b) ^{2}H NMR

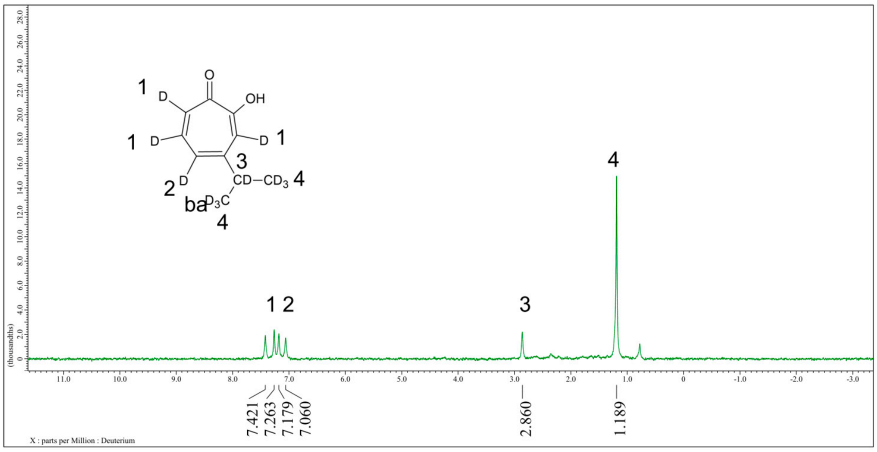

(c) ^{13}C NMR

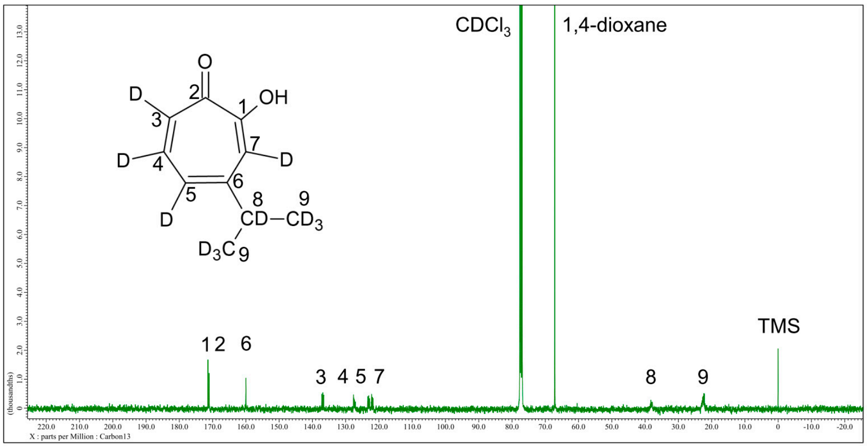

(d) ESI-MS

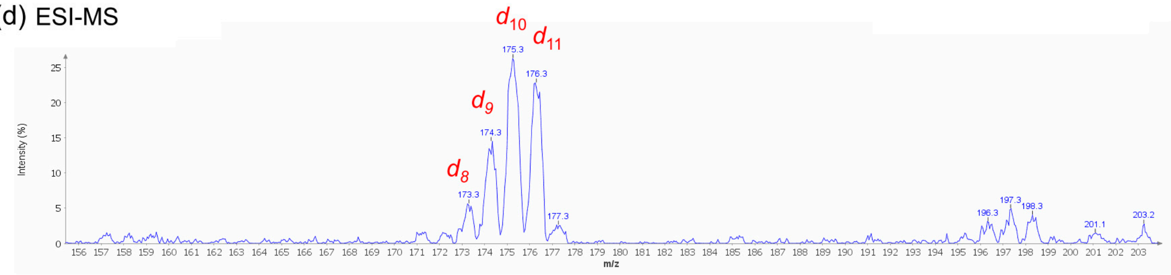

Figure S1. (a) ^{1}H NMR ($CDCl_3$, 400 MHz), (b) ^{2}H NMR ($CHCl_3$, 61.4 MHz), and (c) ^{13}C NMR ($CDCl_3$, 101 MHz) data of hinokitiol-d_{10}. 1,4-Dioxane: deuteration rate reference, $CDCl_3$ or $CHCl_3$: NMR solvents. (d) ESI-MS spectra in positive mode of hinokitiol-d cation showing the mass distribution of the different isotopologues, which ranges from d_{11}–d_{15}. The distribution of the isotopologues is as follows (M^+): 7.4 %, d_8; 19.8 %, d_9; 38.7 %, d_{10}; 34.2 %, d_{11} (calculated deuteration level = 90.9 %).

3. EPMA analysis conducted on test samples

To evaluate the applicability of silica coating on PMMA, a coating agent was applied to a plastic substrate and cured at room temperature (~20°C). The cured sample was subsequently analyzed by electron probe microanalysis (EPMA) to investigate its structural characteristics.

EPMA was performed for O, C, and Si using an electron probe micro-analyzer (EPMA JEM8900, JEOL) at an acceleration voltage of 15.0 kV and an irradiation current of $2.5e^{-8}$ mA to measure the distribution state of elements. Figure S2 shows the elemental (C, O, and Si) mapping of the test sample. It was confirmed through EPMA surface analysis that a thin silica coating had been successfully formed on the plastic plate.

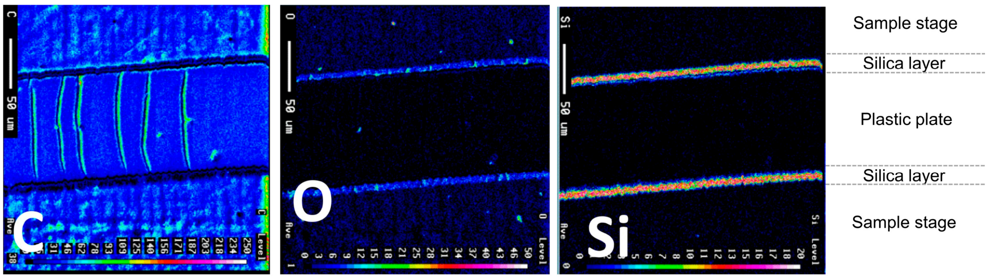

Figure S2. Elemental (C, O, and Si) mapping of the test sample.

4. X-ray and Neutron Reflectivity Analysis

Figure S3 shows the photograph of the SR-HT/PMMA/Si sample (left) and a schematic illustration of its structure.

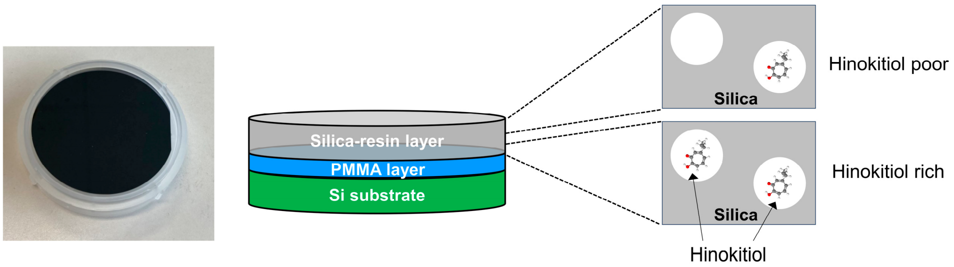

Figure S3. The photograph of the SR-HT/PMMA/Si sample (left) and a schematic illustration of its structure.

Figure S4 shows the X-ray reflectivity (XR) profiles and the fitting results of the air-solid reflectivity data for the SR/PMMA/Si sample. A four-layer model (SR/SR-PMMA mixing layer/PMMA/SiO_2/Si) was employed to fit the obtained XR profiles.

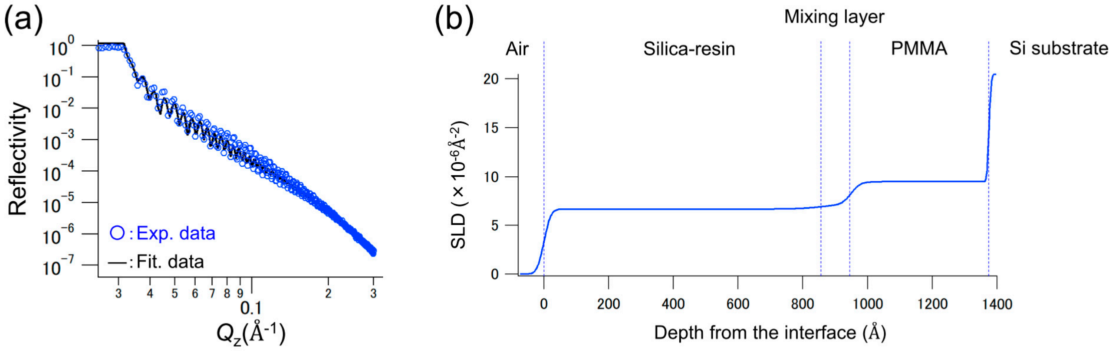

Figure S4. (a) XR profiles of the SR/PMMA/Si sample. The circles represent the experimental data, while the solid lines represent the best-fit calculated NR profiles. (b) X-ray SLD profiles of the SR/PMMA/Si sample calculated from the obtained structural parameters.

Figure S5 shows the neutron reflectivity (NR) profiles and the fitting results of the air-solid reflectivity data for the SR/PMMA/Si sample.

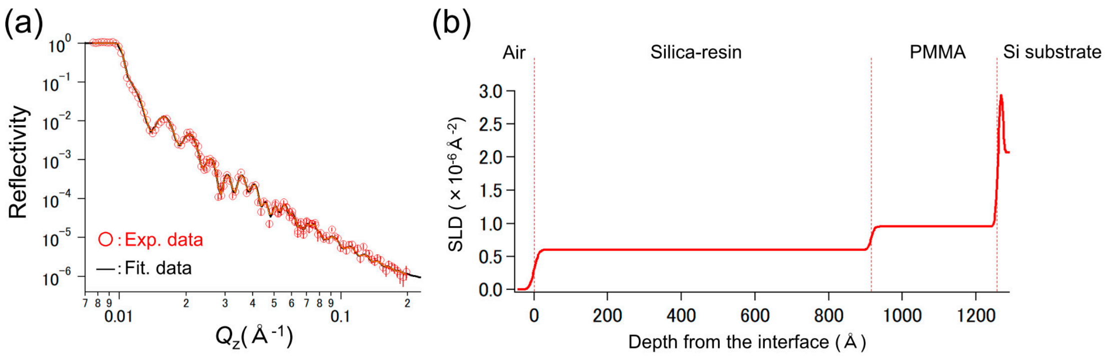

Figure S5. (a) NR profiles of the SR/PMMA/Si sample. The circles represent the experimental data, while the solid lines represent the best-fit calculated NR profiles. (b) Neutron SLD profiles of the SR/PMMA/Si sample calculated from the obtained structural parameters.

Figures S6–12 show the time-dependent NR profiles and the fitting results of the air–solid reflectivity data for the SR/PMMA/Si sample (8 h–91 d).

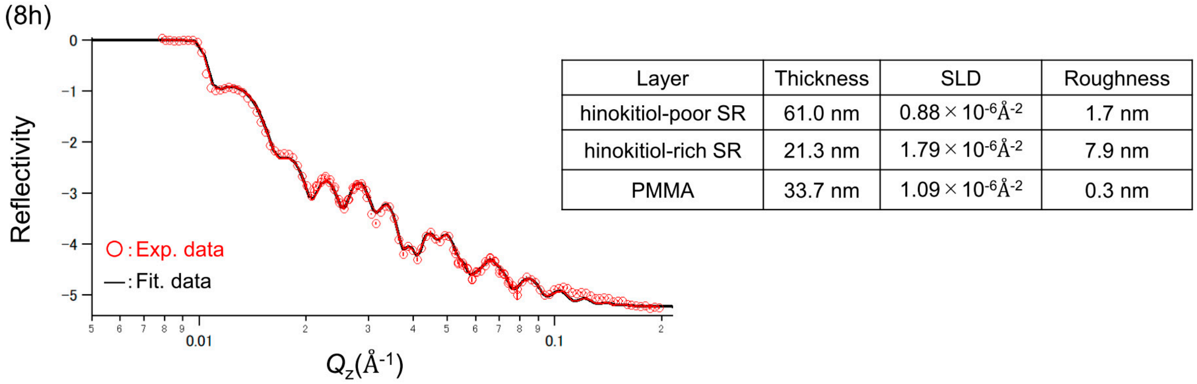

Layer	Thickness	SLD	Roughness
hinokitiol-poor SR	61.0 nm	0.88 × 10^{-6}Å^{-2}	1.7 nm
hinokitiol-rich SR	21.3 nm	1.79 × 10^{-6}Å^{-2}	7.9 nm
PMMA	33.7 nm	1.09 × 10^{-6}Å^{-2}	0.3 nm

Figure S6. NR profiles of the SR/PMMA/Si sample (8 h). The circles represent the experimental data, while the solid lines represent the best-fit calculated NR profiles.

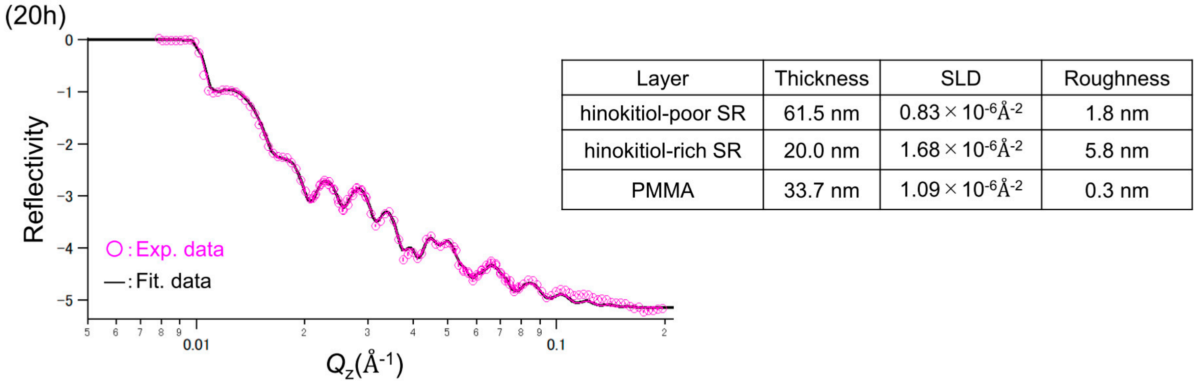

Layer	Thickness	SLD	Roughness
hinokitiol-poor SR	61.5 nm	0.83×10^{-6} Å^{-2}	1.8 nm
hinokitiol-rich SR	20.0 nm	1.68×10^{-6} Å^{-2}	5.8 nm
PMMA	33.7 nm	1.09×10^{-6} Å^{-2}	0.3 nm

Figure S7. NR profiles of the SR/PMMA/Si sample (20 h). The circles represent the experimental data, while the solid lines represent the best-fit calculated NR profiles.

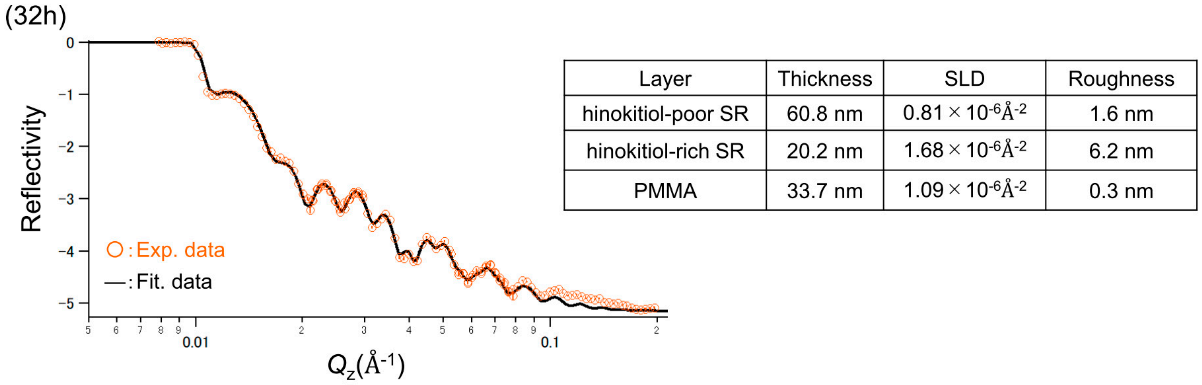

Layer	Thickness	SLD	Roughness
hinokitiol-poor SR	60.8 nm	0.81×10^{-6} Å^{-2}	1.6 nm
hinokitiol-rich SR	20.2 nm	1.68×10^{-6} Å^{-2}	6.2 nm
PMMA	33.7 nm	1.09×10^{-6} Å^{-2}	0.3 nm

Figure S8. NR profiles of the SR/PMMA/Si sample (32 h). The circles represent the experimental data, while the solid lines represent the best-fit calculated NR profiles.

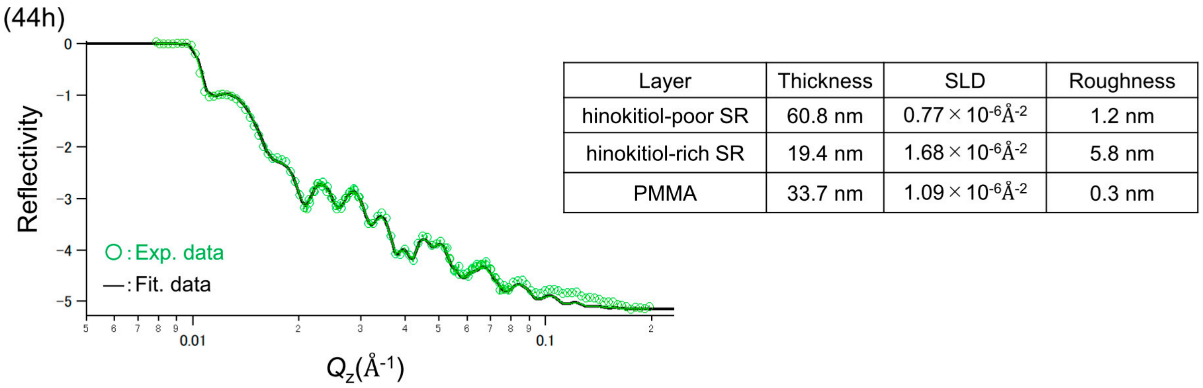

Layer	Thickness	SLD	Roughness
hinokitiol-poor SR	60.8 nm	0.77×10^{-6} Å^{-2}	1.2 nm
hinokitiol-rich SR	19.4 nm	1.68×10^{-6} Å^{-2}	5.8 nm
PMMA	33.7 nm	1.09×10^{-6} Å^{-2}	0.3 nm

Figure S9. NR profiles of the SR/PMMA/Si sample (44 h). The circles represent the experimental data, while the solid lines represent the best-fit calculated NR profiles.

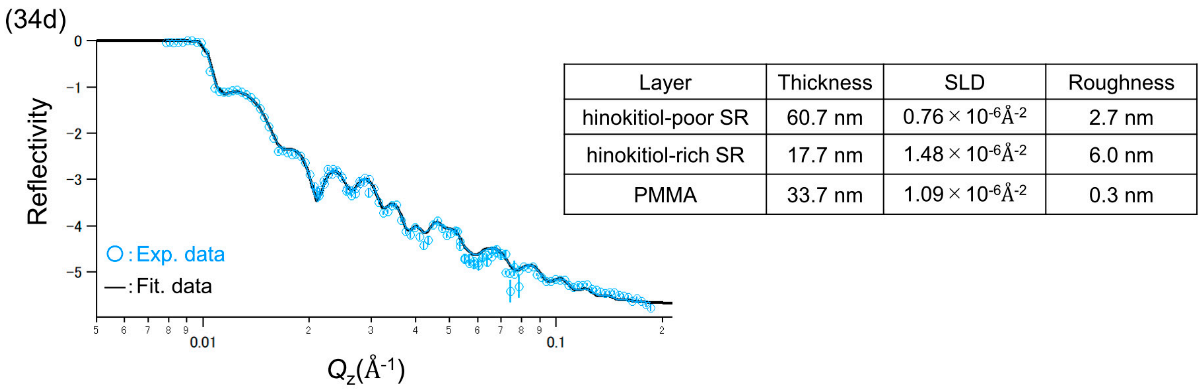

Layer	Thickness	SLD	Roughness
hinokitiol-poor SR	60.7 nm	0.76×10^{-6}Å^{-2}	2.7 nm
hinokitiol-rich SR	17.7 nm	1.48×10^{-6}Å^{-2}	6.0 nm
PMMA	33.7 nm	1.09×10^{-6}Å^{-2}	0.3 nm

Figure S10. NR profiles of the SR/PMMA/Si sample (34 d). The circles represent the experimental data, while the solid lines represent the best-fit calculated NR profiles.

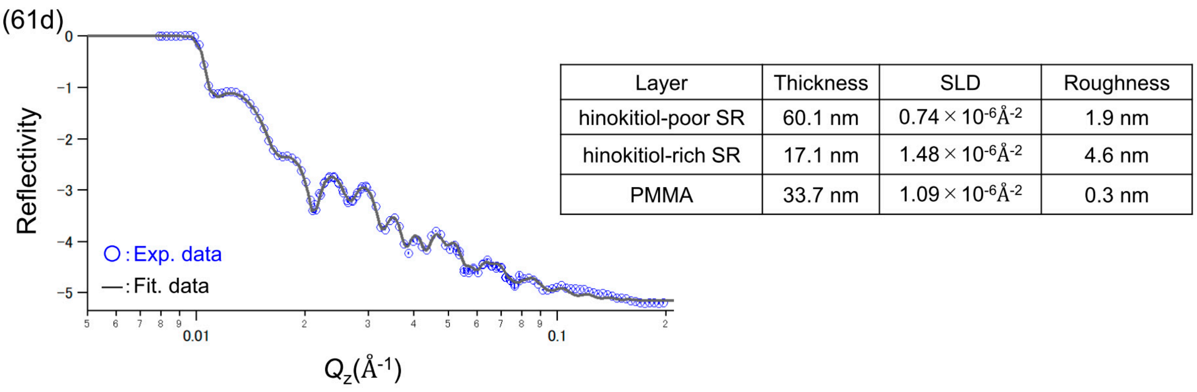

Layer	Thickness	SLD	Roughness
hinokitiol-poor SR	60.1 nm	0.74×10^{-6}Å^{-2}	1.9 nm
hinokitiol-rich SR	17.1 nm	1.48×10^{-6}Å^{-2}	4.6 nm
PMMA	33.7 nm	1.09×10^{-6}Å^{-2}	0.3 nm

Figure S11. NR profiles of the SR/PMMA/Si sample (61 d). The circles represent the experimental data, while the solid lines represent the best-fit calculated NR profiles.

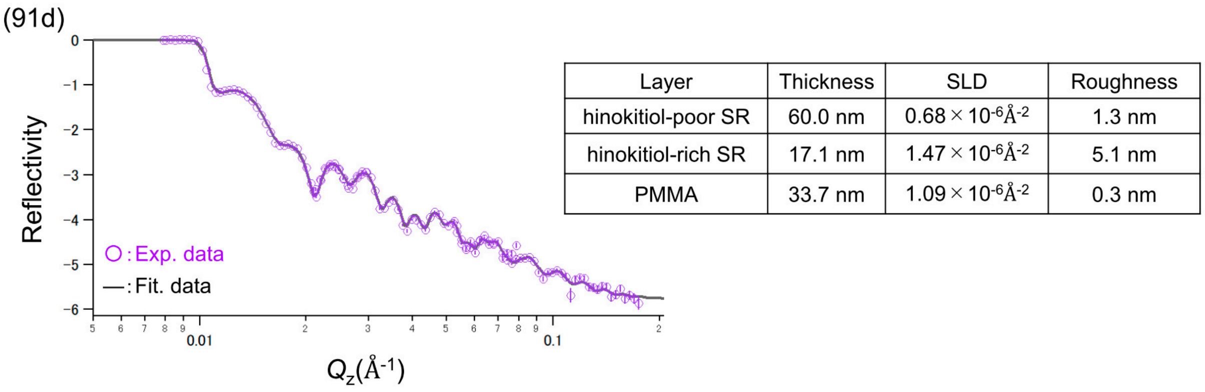

Layer	Thickness	SLD	Roughness
hinokitiol-poor SR	60.0 nm	0.68×10^{-6}Å^{-2}	1.3 nm
hinokitiol-rich SR	17.1 nm	1.47×10^{-6}Å^{-2}	5.1 nm
PMMA	33.7 nm	1.09×10^{-6}Å^{-2}	0.3 nm

Figure S12. NR profiles of the SR/PMMA/Si sample (91 d). The circles represent the experimental data, while the solid lines represent the best-fit calculated NR profiles.

Reference

1. Micheau C, Ueda Y, Akutsu-Suyama K, Bourgeois D, Motokawa R (2023) Deuterated malonamide synthesis for fundamental research on solvent extraction systems. Solv Extr Ion Exch 41:221–240.
